# Decoding the Digital Pulse: Bibliometric Analysis of 25 Years in Digital Health Research Through the Journal of Medical Internet Research

**DOI:** 10.2196/60057

**Published:** 2024-11-15

**Authors:** Robert Kaczmarczyk, Theresa Isabelle Wilhelm, Jonas Roos, Ron Martin

**Affiliations:** 1 Department of Dermatology and Allergy Technical University of Munich Munich Germany; 2 Eye Center—Medical Center Faculty of Medicine Albert-Ludwigs-University of Freiburg Freiburg Germany; 3 Department of Orthopedics and Trauma Surgery University Hospital of Bonn Bonn Germany; 4 Department of Plastic and Hand Surgery, Burn Care Center BG Klinikum Bergmannstrost Halle Halle Germany

**Keywords:** digital health, JMIR publication analysis, network analysis, artificial intelligence, AI, large language models, eHealth, Claude 3 Opus, digital, digital technology, digital intervention, machine learning, natural language processing, NLP, deep learning, algorithm, model, analytics, practical model, pandemic, postpandemic era, mobile phone

## Abstract

**Background:**

As the digital health landscape continues to evolve, analyzing the progress and direction of the field can yield valuable insights. The *Journal of Medical Internet Research* (JMIR) has been at the forefront of disseminating digital health research since 1999. A comprehensive network analysis of JMIR publications can help illuminate the evolution and trends in digital medicine over the past 25 years.

**Objective:**

This study aims to conduct a detailed network analysis of JMIR’s publications to uncover the growth patterns, dominant themes, and potential future trajectories in digital health research.

**Methods:**

We retrieved 8068 JMIR papers from PubMed using the Biopython library. Keyword metrics were assessed using accuracy, recall, and *F*_1_-scores to evaluate the effectiveness of keyword identification from Claude 3 Opus and Gemini 1.5 Pro in addition to 2 conventional natural language processing methods using key bidirectional encoder representations from transformers. Future trends for 2024-2026 were predicted using Claude 3 Opus, Google’s Time Series Foundation Model, autoregressive integrated moving average, exponential smoothing, and Prophet. Network visualization techniques were used to represent and analyze the complex relationships between collaborating countries, paper types, and keyword co-occurrence.

**Results:**

JMIR’s publication volume showed consistent growth, with a peak in 2020. The United States dominated country contributions, with China showing a notable increase in recent years. Keyword analysis from 1999 to 2023 showed significant thematic shifts, from an early internet and digital health focus to the dominance of COVID-19 and advanced technologies such as machine learning. Predictions for 2024-2026 suggest an increased focus on artificial intelligence, digital health, and mental health.

**Conclusions:**

Network analysis of JMIR publications provides a macroscopic view of the evolution of the digital health field. The journal’s trajectory reflects broader technological advances and shifting research priorities, including the impact of the COVID-19 pandemic. The predicted trends underscore the growing importance of computational technology in future health care research and practice. The findings from JMIR provide a glimpse into the future of digital medicine, suggesting a robust integration of artificial intelligence and continued emphasis on mental health in the postpandemic era.

## Introduction

The *Journal of Medical Internet Research* (JMIR), founded in 1999 as the first international scientific peer-reviewed journal focusing on health care research, information, and communication via internet and intranet technologies [1], pioneered several innovations. These included the introduction of new copyright frameworks and the adoption of electronic-only publishing and open-access content [2]. Over the past 25 years, digital health technologies such as telemedicine and wearable biosensors have significantly advanced health care by improving access to information and improving patient monitoring [3-5]. In addition, the use of artificial intelligence (AI) in clinical environments has brought significant advances, such as the ability to automate image analysis. This technology facilitates the early detection of diseases, for example, diabetic retinopathy through retinal images [6,7]. These advances promise to improve personalized medicine, preventative care, and patient outcomes, and the future is likely to continue to be shaped by technological innovation [8,9].

On the occasion of the 25th anniversary of JMIR, we aimed to analyze the development, main topics, and future trends of digital medicine over the past 25 years using the example of JMIR’s publications in a meta-analysis. For this purpose, network analysis serves as an effective tool to process and visualize complex data and to uncover key features and relationships within interconnected systems [10-12]. Key variables impacting a topic can be identified by mapping keywords, countries, or other study-specific elements as nodes, with their connections as edges in a network. This visualization helps systematically uncover core themes and reveal influential factors and interactions among various research elements. This approach has been effectively applied in dermatological research to analyze trends through congress data [13,14]. This method enriches our understanding of the structure and evolution of a research field and provides new insights into how individual studies contribute to the broader scientific discourse.

In this study, we therefore conducted a comprehensive network analysis of JMIR’s publications over the last quarter century to illuminate the evolution, prevailing trends, and future paths of digital health. By thoroughly examining paper types, paper titles, countries, MeSH (Medical Subject Headings) terms, and keywords, we constructed a complex picture of the digital health landscape, highlighting key themes, breakthroughs, and the global distribution of papers. The method aimed to identify critical junctures in the development of digital health, including the rise of telemedicine, the proliferation of mobile health apps, and the introduction of AI. With this, we provide a comprehensive overview of the evolution of the field and insights into emerging trends that will redefine the future of health care.

## Methods

### Ethical Considerations

This study exclusively used publicly accessible, secondary data from internet sources (PubMed) and did not involve human participants or any sensitive personal information. As such, it did not require ethics board approval.

### Data Acquisition

We conducted a comprehensive review of papers published over 25 years (1999-2023) in JMIR, which is fully indexed by PubMed. Using the Biopython (version 1.83; Bioinformatics Open Source Project [15]) library, we retrieved a dataset of 8068 papers as of March 20, 2024, using the search query “(“Journal of Medical Internet Research”[Journal])” and the first entry in the PubMed history as the reference year for the following analyses.

### MeSH Terms and Keyword Analysis

Following the initial exploratory data visualization, we encountered challenges due to incomplete data and a high frequency of generic terms like “Humans” within the MeSH terms (Multimedia Appendix 1). Consequently, Anthropic’s most advanced large language model, Claude 3 Opus (version 20240229 [16]) with the Anthropic Python application programming interface library (version 0.19.1 [17]) as well as Gemini 1.5 Pro (February 2024) using Google’s Generative-AI Python application programming interface library [18], was used to derive keywords based on the paper titles and abstracts. We prompted the model to generate a concise JSON list of 6 to 12 significant keywords for each paper. Keywords were adjusted to be concise and in Title Case, as specified by the prompt requirements (Multimedia Appendix 2).

### Evaluation of Keyword Metrics

We assessed keyword quality from several sources, including PubMed MeSH terms, author keywords, Claude 3 Opus keywords, Gemini 1.5 Pro (February 2024), and 2 conventional natural language processing (NLP) methods using key bidirectional encoder representations from transformers [19] with the scientific NLP models en_core_sci_lg and en_core_sci_md [20], against the reference derived from paper titles and abstracts in addition to the MeSH terms and author keywords or the MeSH terms and author keywords alone. All text data were converted to lowercase for case-insensitive matching. Keywords were aggregated into a unified set, and presence or absence matrices were constructed for each source. Precision, recall, and *F*_1_-score were calculated to evaluate the effectiveness of each source in identifying keywords.

### Statistical Analysis

To account for fluctuations in the number of papers published each year, we first calculated the annual percentage of keyword occurrences and subsequently computed the average of these annual percentages for each defined period (1999-2007, 2008-2013, 2014-2019, and 2020-2023). This method ensures that years with fewer publications do not disproportionately affect the analysis within each period. The time frames were arbitrarily chosen based on initial exploratory analysis and took into consideration the rising number of publications, which justified the inclusion of more years in earlier time frames. Additionally, the onset of the COVID-19 pandemic in 2020 represented a natural turning point.

### Future Trend Prediction

To evaluate the performance of different keyword trend prediction methods, we used various metrics including mean absolute error (MAE), mean squared error (MSE), root-mean-squared error (RMSE), *R*^2^, and mean absolute percentage error (MAPE). The models assessed were Claude 3 Opus, autoregressive integrated moving average (ARIMA), exponential smoothing (ES), Facebook’s Prophet, and the Foundation Model Time Series Foundation Model (TimesFM) by Google and tested against the actual keywords (MeSH+author keywords set) for the first 7 months of 2024.

### Network Visualization

Network visualization was performed using Gephi (version 0.10.1; Gephi Consortium [21]). Co-occurrence matrices were generated for keywords appearing together in a paper, for combined assignments of different paper types to a paper, and for collaborating locations contributing to a paper. The generation of co-occurrence matrices was done by importing CSV-based data of keywords, paper types, or contributing countries into Gephi, recognizing edges between entities that co-occur on a line-by-line basis, that is, in a paper. Nodes were thus represented by the keywords, paper types, or contributing countries. Edges corresponded to the co-occurrence of nodes within a paper. Node size and labeling were proportional to node frequency. Edge strength, as indicated by the line thickness between 2 nodes, was set in relation to the weight of the edge, that is, the co-occurrence frequency. To ensure readability, we implemented a minimum keyword frequency filter ranging from 3 to 30, depending on the total number of keywords in the 4 subsets of years (see figure descriptions). The network compositions were created using the Fruchterman-Reingold layout [22] and the ForceAtlas algorithm [23]. While the Fruchterman-Reingold layout excels at providing a balanced distribution and minimizing edge crossings, making it suitable for intuitive and interpretable visualization of connected nodes in close proximity, the ForceAtlas layout effectively emphasizes community structures and efficiently handles large networks, making it particularly effective for dense and complex network visualizations. We therefore applied the Fruchterman-Reingold layout to visually cluster related keywords and paper types, where its ability to maintain readability and coherence was critical, and used the ForceAtlas to visualize the network of contributing countries, where the algorithm’s strength in cluster detection was particularly useful in revealing the underlying community structure. To ensure readability and optimize visualization, network parameters were manually adjusted, including minimum node size (10.0), maximum node size (100.0), attraction force (15.0), area (10,000.0), node opacity (70.0), edge thickness (3.0), and edge opacity (15.0) for the Fruchterman-Reingold graphs and repulsion strength (200.0), attraction strength (20.0), maximum displacement (10.0), auto-stabilization strength (80.0), attraction force (30.0), node opacity (1000.0), edge thickness (3.0), and edge opacity (30.0) for the ForceAtlas graph. Edges were colored to match the nodes they connected, based on the determined modularity class. Nodes in the same class were assigned the same color. Modularity, which measures the ability of the network to partition into distinct communities [24], was used for this determination.

## Results

### Publication Trends and Global Contribution Analysis in JMIR

JMIR has experienced significant publication growth over the past 25 years, starting with 15 papers in 1999 and reaching a peak of 1567 in 2020. The journal consistently increased its publication volume each year, exceeding 100 papers per year since 2011. However, there was a decline to 491 papers in 2023. Our analysis included 8068 papers, with an average presence of 9 (SD 4.2) MeSH terms and 6.5 (SD 4.3) keywords per paper. Notably, 212 (2.6%) papers had no MeSH terms, and 1012 (12.5%) papers had no keywords.

**Figure 1:**
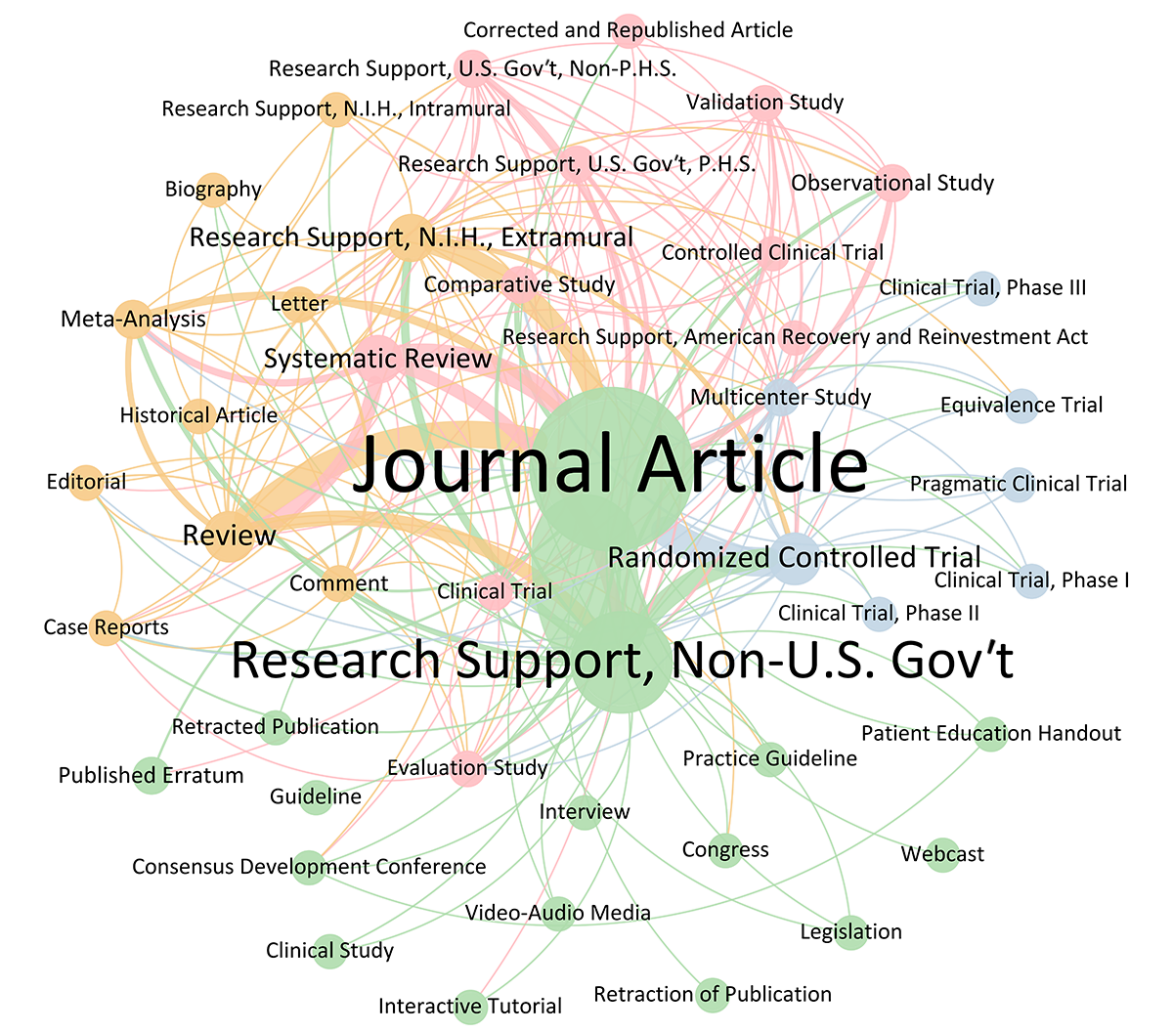
Weighted network visualization (Fruchterman-Reingold layout) of all paper types from 1999 to 2023 corresponding to 44 nodes and 205 edges and a modularity of 0.157 with 4 communities found (nodes of the same color correspond to the same community).

Our review of paper types in the publication database reveals that “Journal Article” is the most common category (n=7806, 96.8%), followed by “Research Support, Non-U.S. Gov’t” (n=4069, 50.4%), “Randomized Controlled Trials” (n=1076, 13.3%), “Review” (n=987, 12.2%), and “Systematic Reviews” (n=824, 10.2%). In combined article classifications, “Journal Article” paired with “Research Support, Non-U.S. Gov’t” is most frequent (n=2109, 26.1%), followed by “Journal Article” alone (n=1995, 24.7%; Figure 1).

Over the years, the distribution of scientific paper contributions by country exhibited significant changes (Table 1). From 1999 to 2007, Canada was in the lead (n=41, 12.8%), followed by the United States (n=18, 5.6%). In the period of 2008-2013, the United States took the lead (n=172, 18.1%, +12.5%), and in 2014-2019, its contribution increased (n=1059, 26.5%, +8.4%). The most recent data from 2020 to 2023 show the United States with a reduced share (n=1378, 20.1%, –6.4%), while China’s participation increased (n=609, 8.9%, +3.9%). The United Kingdom (top 2 to 7) and Australia (top 3 to 4) remained consistently among the top contributors over all periods. Emerging contributors such as Singapore (n=119, 1.7%) and Hong Kong (n=140, 2%) highlight the expanding global diversity in research authorship.

**Figure 2:**
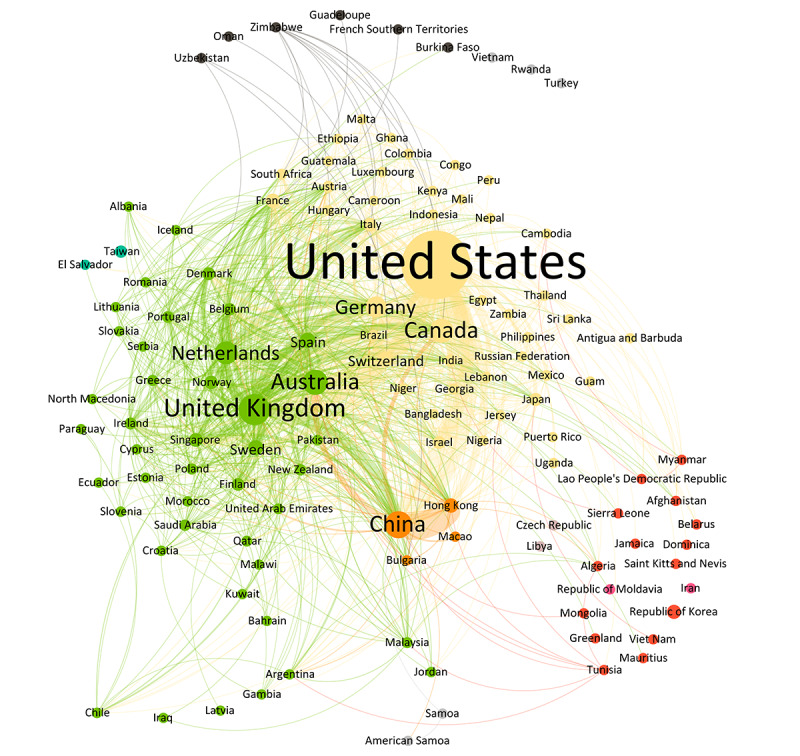
Weighted network visualization (ForceAtlas layout) of country contributions from 1999 to 2023 corresponding to 125 nodes and 1055 edges and a modularity of 0.112 with 9 communities found (countries of the same color correspond to the same community).

The network analysis of the contributing countries over the entire period from 1999 to 2023 delineates the pivotal role of regions characterized by sustained high levels of scientific output and robust collaborations with other regions. Notably, countries such as the United States, the United Kingdom, and China emerge as central nodes within clusters of identical modularity classes. Meanwhile, peripheral nations such as Australia assume a mediating function, attributable to their close affiliations with adjacent clusters, exemplified by strong connections to the United States, China, and Europe (Figure 2).

**Table 1:** Trends in country contribution to JMIR publications (1999-2023)^a^.

1999-2007 (n=319)		2008-2013 (n=933, +614, +129.5%)				2014-2019 (n=2864, +1931, +207%)				2020-2023 (n=3952, +1088, +38%)		
Country	Values, n (%)	Country	Values, n (%)	Trend (%)	Country		Values, n (%)	Trend (%)	Country		Values, n (%)	Trend (%)
Canada	41 (12.8)	United States	172 (18.1)	+12.5	United States		1059 (26.5)	+8.4	United States		1378 (20.1)	–6.4
United States	18 (5.6)	Netherlands	122 (12.9)	+8.5	United Kingdom		407 (10.2)	+3.2	China		609 (8.9)	+3.9
Netherlands	14 (4.4)	Australia	80 (8.4)	+5	Australia		306 (7.7)	–0.8	United Kingdom		534 (7.8)	–2.4
Australia	11 (3.4)	Canada	76 (8)	–4.8	Netherlands		250 (6.3)	–6.6	Australia		335 (4.9)	–2.8
Germany	10 (3.1)	United Kingdom	66 (7)	+4.5	Canada		224 (5.6)	–2.4	Canada		330 (4.8)	–0.8
Denmark	8 (2.5)	Sweden	37 (3.9)	+3.3	China		198 (5)	+3.5	Germany		308 (4.5)	0
United Kingdom	8 (2.5)	Germany	24 (2.5)	–0.6	Germany		181 (4.5)	+2	Netherlands		237 (3.5)	–2.8
Greece	7 (2.2)	Norway	22 (2.3)	+0.5	Switzerland		88 (2.2)	+0.1	Switzerland		168 (2.4)	+0.2
Norway	6 (1.9)	Switzerland	20 (2.1)	+0.6	Sweden		83 (2.1)	–1.8	Spain		149 (2.2)	+0.2
Switzerland	5 (1.6)	Japan	14 (1.5)	+1.2	Spain		77 (1.9)	+0.7	Hong Kong		140 (2)	+0.7
Spain	4 (1.2)	China	14 (1.5)	+1.2	Norway		66 (1.7)	–0.7	France		133 (1.9)	+0.4
New Zealand	3 (0.9)	Spain	12 (1.3)	0	France		61 (1.5)	+1	Singapore		119 (1.7)	+0.3
Italy	3 (0.9)	Finland	10 (1.1)	New	Singapore		57 (1.4)	New	Sweden		113 (1.6)	–0.4
Nigeria	2 (0.6)	Italy	10 (1.1)	+0.1	Hong Kong		54 (1.4)	+0.6	Italy		102 (1.5)	+0.6
Niger	2 (0.6)	Hong Kong	7 (0.7)	New	Denmark		51 (1.3)	+0.6	Japan		82 (1.2)	+0.3

^a^This table displays the distribution and trends of country contributions to the JMIR over 4 time periods. The countries are listed in descending order based on the number of publications (n) for each period accompanied by their corresponding percentage (%) of the total publications within that time frame and the trend that is indicating the percentage point change compared to the previous period. Significant shifts include the increasing dominance of the United States, a marked rise in publications from China in the most recent period, and the consistent presence of countries such as the United Kingdom, Australia, and Canada throughout the 25 years.

### Keyword Benchmark

The comparative analysis of keyword sets from different sources highlighted variations in accuracy, recall, and *F*_1_-scores. The Claude 3 Opus keyword set demonstrated the highest accuracy (0.84) and *F*_1_-score (0.23), suggesting effective keyword identification compared to Gemini 1.5 Pro and conventional NLP (Table 2). However, it is important to note that these metrics are based on keyword occurrences in at least 1 of the MeSH, keywords, paper titles, or abstract sets, which is only a rough estimate of the true keyword relevance. Despite this limitation, the comprehensive coverage and consistent performance of the extracted Claude 3 Opus keywords and the absence of missing data led to its selection for further analysis.

**Table 2:** Evaluation of keyword quality across different methods and sources^a^.

Category and method		n=7904 (98%)^b^						n=8068 (100%)		
		Reference: MeSH+author keywords^c^+titles+abstracts^d^	Reference: MeSH+author keywords^c^				Keywords per paper, mean (SD)			Words per keyword, mean (SD)
		Accuracy	Accuracy	Recall	*F*_1_-score					
**Reference**										
	MeSH+keywords	1.00	1.00	1.00	1.00	14.52 (5.63)			1.70 (0.38)	
**Reference (split)**										
	MeSH	0.99	0.99	0.64	0.78	9.00 (4.20)			1.63 (0.40)	
	Keywords	0.89	0.89	0.44	0.59	6.51 (4.34)			1.61 (0.79)	
**Large language model**										
	Claude 3 Opus keywords	0.84	0.28	0.20	0.23	9.83 (1.29)			1.82 (0.27)	
	Gemini 1.5 Pro keywords	0.72	0.19	0.14	0.16	9.35 (3.26)			1.87 (0.63)	
**Conventional NLP^e^ using KeyBERT^f^**										
	spacy-en_core_sci_md	0.80	0.00	0.00	0.00	9.00 (0.07)			2.89 (0.14)	
	spacy-en_core_sci_lg	0.80	0.00	0.00	0.00	9.00 (0.07)			2.88 (0.14)	

^a^Comparison of keyword quality of various methods and sources, including PubMed MeSH terms, author keywords, large language model–generated keywords (Claude 3 Opus and Gemini 1.5 Pro), and conventional NLP methods (using KeyBERT with different models). The reference set is constructed from MeSH terms, author keywords, titles, and abstracts or only MeSH terms and author keywords. The metrics presented include accuracy, precision, recall, and *F*_1_-score. Additionally, the table provides the mean and SD of the number of keywords per paper and the mean number of words per keyword. Claude 3 Opus demonstrates superior performance with higher accuracy (0.84) and *F*_1_-score (0.23) compared to Gemini 1.5 Pro and conventional keyword extraction using KeyBERT.

^b^A total of 7904 (98%) represents the number of papers with available reference categories (MeSH terms, author keywords, or both). This subset was used for calculating accuracy, precision, recall, and *F*_1_-metrics.

^c^For author keywords, an exact case-insensitive string match between the generated keyword and any item in the reference list (MeSH terms or author keywords) was considered correct.

^d^For titles and abstracts, a keyword was considered correct if it appeared as a case-insensitive substring within the paper’s title or abstract text.

^e^NLP: natural language processing.

^f^KeyBERT: key bidirectional encoder representations from transformers.

### Keyword Trend Analysis With Claude Opus 3 (1999-2023)

**Figure 3:**
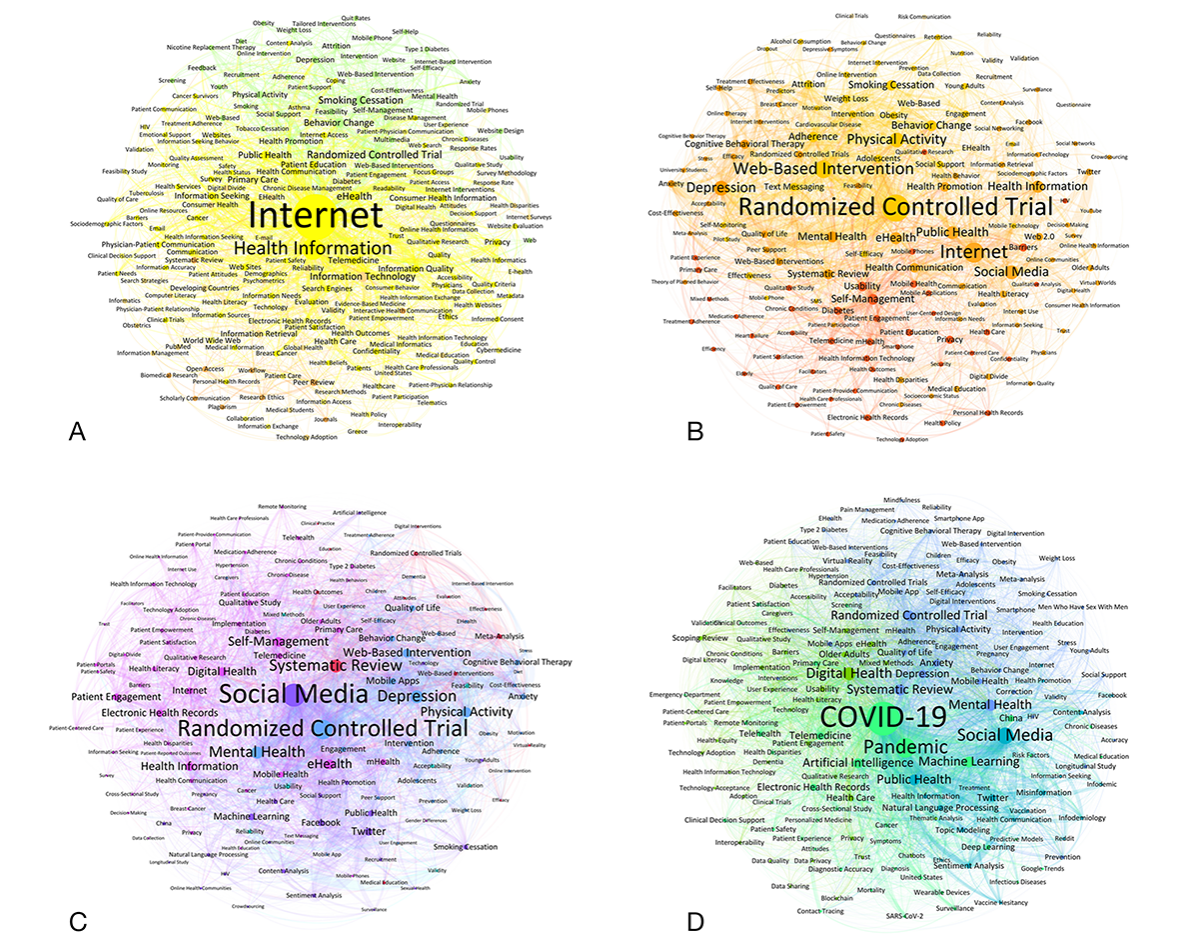
Weighted network visualization (Fruchterman-Reingold layout) of the most frequent paper keywords in the 4 periods (A) 1999-2007, (B) 2008-2013, (C) 2014-2019, and (D) 2020-2023 with a minimum keyword frequency of (A) 3, (B) 9, (C) 25, and (D) 30. This corresponded to (A) 1675 nodes (n=201, 12% visible), 11,600 edges (n=1624, 14% visible), and 5 found communities with a modularity of 0.32; (B) 31,400 nodes (n=157, 0.5% visible), 69,900 edges (n=2796, 4% visible), and 5 found communities with a modularity of 0.28; (C) 13,900 nodes (n=139, 1% visible), 113,575 edges (n=4543, 4% visible), and 5 found communities with a modularity of 0.2; and (D) 16,300 nodes (n=163, 1% visible), 147,575 edges (n=5903, 4% visible), and 4 found communities with a modularity of 0.2.

The keyword analysis from 1999 to 2023 showed significant thematic shifts across 4 periods (Table 3):

- 1999-2007 (n=319 papers): The primary keywords are “Internet” (n=121, 41.9%) and “Health Information” (n=50, 16.7%), followed by “Randomized Controlled Trial” (n=20, 5.2%) and “eHealth” (n=18, 5%), highlighting early focuses on basic internet applications in health care (Figure 3A).
- 2008-2013 (n=933 papers): Trending keywords comprise “Randomized Controlled Trial” (n=129, 13.9%, +8.7%), “Physical Activity” (n=62, 7.6%, +5.4%), “Web-Based Intervention” (n=78, 7.6%, +5.8%), and “Depression” (n=62, 6.9%, +4.3%), whereas the more generic term “Internet” (n=91, 12.3%, –29.5%) is on the decline (Figure 3B).
- 2014-2019 (n=2864 papers): “Social Media” (n=356, 12.8%, +8.9%) and “Randomized Controlled Trial” (n=302, 11.1%, –2.8%) were the most extracted keywords, though the latter with a slight decrease. “Systematic Review” (n=197, 6.7%, +3.4%) and “Mental Health” (n=183, 6%, +1.9%) are under the trending keywords (Figure 3C).
- 2020-2023 (n=3952 papers): The impact of the pandemic led to “COVID-19” (n=980, 22.9%, +22.9%) being the most prominent keyword, accompanied by related terms like “Pandemic” (n=559, 12.8%, +12.8%), “Social Media“ (n=460, 11.7%, –1.1%), and “Digital Health” (n=350, 9.4%, +5.5%; Figure 3D). The integration of advanced technology in medical research in the form of “Artificial Intelligence” is on the rise (n=259, 7.6%, +6.6%; Figure 3D).

**Table 3:** Future trend prediction benchmark for the year 2024 across several methods^a^.

Model	MAE	MSE	RMSE	MAPE
Claude 3 Opus	*1.85* ^b^	*8.63*	*2.94*	80.09
TimesFM	1.86	9.24	3.04	*79.03*
ARIMA	1.90	8.85	2.98	82.36
ES	1.96	9.03	3.01	86.13
Prophet	4.41	217.90	14.76	232.30

^a^This table presents the benchmark results of various models for future keyword trend prediction. The performance metrics include mean absolute error (MAE), mean squared error (MSE), root-mean-squared error (RMSE), and mean absolute percentage error (MAPE). Claude 3 Opus, Time Series Foundation Model (TimesFM), autoregressive integrated moving average (ARIMA), exponential smoothing (ES), and Prophet were evaluated against the actual keyword data (the MeSH and author keywords are considered ground truth) for the first 7 months of 2024. Claude 3 Opus exhibited the lowest errors across most metrics, indicating its superior accuracy in predicting keyword trends.

^b^Best results are marked in italics format.

### Future Trend Prediction Benchmark

Claude 3 Opus achieved the lowest MAE at 1.85, the lowest MSE at 8.62, and the lowest RMSE at 2.94. TimesFM showed the best performance in terms of MAPE with a value of 79.03, slightly outperforming Claude 3 Opus in this metric. ARIMA, ES, and Prophet exhibited higher errors across all metrics compared to Claude 3 Opus and TimesFM. Specifically, Claude 3 Opus demonstrated significant improvements over other models. Compared to ARIMA, Claude 3 Opus showed a 2.59% improvement in MAE, a 2.55% improvement in MSE, a 1.28% improvement in RMSE, and a 4.04% improvement in MAPE. When compared to ES, Claude 3 Opus had a 5.59% improvement in MAE, a 4.50% improvement in MSE, a 2.28% improvement in RMSE, and an 8.24% improvement in MAPE. The Prophet model lagged considerably behind, with Claude 3 Opus showing improvements of 57.98% in MAE, 96.04% in MSE, 80.10% in RMSE, and 65.98% in MAPE. Although TimesFM performed slightly better in terms of MAPE, Claude 3 Opus still showed a minimal 0.61% improvement in MAE, a 6.65% improvement in MSE, and a 3.38% improvement in RMSE over TimesFM (Table 3).

### Predicted Keyword Trends by Claude Opus 3 (2024-2026)

For the period from 2024 to 2026, we have leveraged Claude 3 Opus, which emerged as the top-performing model in 3 of 4 categories in our 2024 future trend prediction benchmark (Table 3). Claude 3 Opus predictions indicate an increased focus on “Artificial Intelligence” (17.8%, +10.2%) and “Digital Health” (13.2%, +3.8%), as also seen in the trending keywords “ChatGPT” (11.8%, +10.6%), “GPT-4” (7.8%, +7.4%), “Large Language Models” (9.1%, +8.3%), and “Natural Language Processing” (7.5%, +3.7%). There is a further reduction of “COVID-19” specific research (10.2%, –12.7%). “Public Health” is expected to decline (5.6%, –1.1%; Table 4).

**Table 4:** Evolution of keyword prevalence in JMIR publications (1999-2026)^a^.

Claude 3 Opus–derived keywords from PubMed paper titles and abstracts																Prediction		
1999-2007 (n=319)			2008-2013 (n=933)				2014-2019 (n=2864)				2020-2023 (n=3952)				2024-2026^b^			
Keyword list	Values, n (%)	Keyword list		Values, n (%)	Trend (%)	Keyword list		Values, n (%)	Trend (%)	Keyword list		Values, n (%)	Trend (%)	Keyword list			Values, %	Trend (%)
													
Internet	121 (41.9)	Randomized Controlled Trial		129 (13.9)	8.7	Social Media		356 (12.8)	8.9	COVID-19		980 (22.9)	22.9	Artificial Intelligence			17.8	+10.2
Health Information	50 (16.7)	Internet		91 (12.3)	–29.5	Randomized Controlled Trial		302 (11.1)	–2.8	Pandemic		559 (12.8)	12.8	Digital Health			13.2	+3.8
Randomized Controlled Trial	20 (5.2)	Physical Activity		62 (7.6)	5.4	Depression		196 (6.8)	–0.1	Social Media		460 (11.7)	–1.1	Machine Learning			12.7	+5.1
eHealth	18 (5)	Web-Based Intervention		78 (7.6)	5.8	Systematic Review		197 (6.7)	3.4	Digital Health		350 (9.4)	5.5	Social Media			12.1	+0.4
Information Quality	12 (4.8)	Depression		62 (6.9)	4.3	Mental Health		183 (6)	1.9	Systematic Review		288 (7.6)	0.9	ChatGPT			11.8	+10.6
Information Technology	17 (4.7)	eHealth		47 (6.8)	1.8	Physical Activity		150 (5.8)	–1.8	Machine Learning		281 (7.6)	4.6	COVID-19			10.2	–12.7
Public Health	14 (4.5)	Health Information		48 (5.8)	–10.9	eHealth		149 (5.3)	–1.5	Artificial Intelligence		259 (7.6)	6.6	Systematic Review			9.6	+2
Smoking Cessation	18 (4.4)	Public Health		52 (5.2)	0.7	Web-Based Intervention		132 (5.1)	–2.5	Mental Health		296 (7.4)	1.4	Large Language Models			9.1	+8.3
World Wide Web	8 (3.9)	Behavior Change		41 (5.1)	1.5	Self-Management		146 (5)	1.2	Randomized Controlled Trial		272 (7.3)	–3.8	Randomized Controlled Trial			8.5	+1.2
Information Retrieval	10 (3.9)	Smoking Cessation		39 (4.5)	0.1	Health Information		126 (4.7)	–1	Public Health		285 (6.7)	3.4	Mental Health			8.2	+0.8
Telemedicine	12 (3.9)	Health Promotion		34 (4.3)	1.7	Twitter		115 (4.4)	3.2	Depression		206 (5.4)	–1.3	GPT-4			7.8	+7.4
Primary Care	13 (3.9)	Mental Health		42 (4.1)	2.3	Internet		99 (4.2)	–8.2	Telemedicine		186 (4.5)	1.7	Natural Language Processing			7.5	+3.7
Behavior Change	15 (3.6)	Web 2.0		25 (4)	+4	Digital Health		127 (3.9)	3	Electronic Health Records		166 (4.3)	0.7	Depression			7.1	+1.7
Patient Education	11 (3.2)	Social Media		57 (3.9)	+3.9	Facebook		92 (3.7)	2.6	Anxiety		171 (4.2)	1.4	Health Care			6	+2.1
Ethics	8 (3.2)	Health Communication		34 (3.8)	1.7	Patient Engagement		105 (3.6)	1.3	Twitter		159 (4.1)	–0.3	Public Health			5.6	–1.1
Health Care	10 (3.2)	Self-Management		40 (3.8)	1.8	Electronic Health Records		109 (3.5)	1.2	Health Care		142 (3.9)	2.1	Microsoft Bing Chat			5.2	+5.1
Privacy	10 (3.2)	Usability		34 (3.7)	3	Mobile Apps		97 (3.4)	3	Natural Language Processing		132 (3.8)	2.4	Telehealth			5	+1.9
Information Seeking	11 (3.1)	Adherence		38 (3.7)	2.3	Behavior Change		89 (3.3)	–1.7	China		146 (3.3)	1.9	Deep Learning			4.8	+2
Consumer Health Information	10 (2.8)	Web-Based Interventions		24 (3.6)	2.1	Public Health		89 (3.3)	–1.9	Physical Activity		134 (3.3)	–2.5	Anxiety			4.7	+0.5
Search Engines	8 (2.7)	Obesity		26 (3.4)	2.7	Intervention		88 (3.2)	0.8	Older Adults		126 (3.2)	1.1	Electronic Health Records			4.7	+0.4

^a^This table presents a longitudinal analysis of keyword prevalence (generated from paper titles and abstracts by Claude 3 Opus) in the JMIR across 5 distinct periods from 1999 to a predictive analysis for 2024-2026. For each period, the top-ranking keywords are listed by their occurrence frequency (n), alongside the mean percentage (%) of papers they were featured in. The keywords are listed in descending order based on the mean percentage of keyword occurrence. Trends are calculated as the percentage point change from the previous period, reflecting shifts in research focus. Key observations include the initial prominence of “Internet,” which gave way to “Randomized Controlled Trial” and later to “COVID-19” during the pandemic, with “Artificial Intelligence” predicted to lead in the coming years.

^b^For the 2024-2026 period, no occurrence frequency (n) is provided as Claude 3 Opus, without the knowledge of the total frequency, only provided a relative distribution in percentages.

## Discussion

### Principal Findings

The main objective of this study was to analyze the digital transformation in the field of medicine over the last 25 years using JMIR as an example. Our analysis offers a detailed view of the journal’s growth and the shifting focus of its papers.

JMIR has seen a steady increase in publications annually, peaking at 1567 papers in 2020. This trend underscores the journal’s expanding role in the digital health field and aligns with the broader rise in digital medicine technologies and applications, particularly from 2010 onward with advances in digital health and mobile health [25]. The surge in 2020 is attributed to the COVID-19 pandemic, which spurred an urgent expansion in digital health solutions like remote monitoring [26] as well as general changes in handling medical research funding, for example, “research community mobilized to submit and review grants more rapidly than ever before” [27].

The peak reflects JMIR’s pivotal role in publishing timely research during this crisis. The decline in papers by 2023 suggests a possible stabilization or emerging challenges in the field, such as funding variations or research topic saturation, a trend that is also becoming apparent globally. For example, the number of publications in PubMed under the search terms eHealth [28] or mobile health [29] also shows a peak value in 2021 with a continuous decline since then. Another possible reason could be the flood of publications on COVID-19 as a global focus of interest. Currently, there is a lack of scientific studies offering explanations for this phenomenon.

### Emergence of Internet and eHealth in Medical Research (1999-2007)

During the early years of the JMIR, the focus was primarily on the broad use of the internet in health contexts, evident from “Internet” being the most frequent keyword. There was a strong emphasis on the provision and dissemination of health information (“Health Information”) and the use of the internet for interventions, as indicated by the presence of keywords like “Smoking Cessation” and “Behavior Change.” Several studies of the population’s use of the internet for health information have been conducted during this period [30-32]. The use of the internet for medical research has also already been scientifically investigated, and early warnings have been given about the consequences that problems can become worse [33]. Nowadays, it is controversial, but it has been shown that internet research can lead to a better understanding of symptoms and diagnoses [34].

In particular, the use of questionnaires as a scientific tool was discussed, and it was critically noted that the population still must familiarize itself with the use of the internet [35,36]. As a result, the term eHealth appears and seeks a further definition [37]. Whereas today, it is defined as a set of technologies applied via the internet to improve quality of life and facilitate health care [38]. This shows the beginnings of digital medicine and scientific research. Working titles such as “The WWW of the World Wide Web: Who, What, and Why?” show the demands on science but also the confrontation with the new topic [39]. Additionally, terms such as “Information Technology” emphasize the technological perspective. This era marks the foundational phase of digital health, characterized by the initial exploration of the internet’s applications in health care and patient education.

### Behavioral Health and eHealth Expansion (2008-2013)

This era marked a notable shift toward more targeted applications and methodologies in the realm of digital health research. The prominence of “Randomized Controlled Trial” as a leading keyword underscores an intensifying commitment to rigorous scientific evaluation of digital health interventions. The rise of “Web-Based Intervention” and “Social Media” as key terms highlights the broadening of platforms and approaches used in health initiatives, suggesting a move toward more web-based and user-engaged strategies. Especially various works on the use of smartphones show a trend toward individualized digital medicine [40-42]. The decline in the dominance of the “Internet” keyword (from n=121, 41.9% in 1999-2007 to n=91, 12.3%, –29.5% in 2008-2013) indicates its ubiquitous presence rather than a novel aspect to be noted. During this period, mental health received increased attention, with “Depression” emerging as a significant focus area, emphasizing the expansion of digital strategies to address mental health issues.

### Social Media, Mental Health Focus, and Systematic Reviews (2014-2019)

The focus shifted significantly toward social media and systematic reviews, indicating a growing recognition of these platforms’ impact on public health and research dissemination. Keywords like “Digital Health” and “Electronic Health Records” suggest a deeper integration of advanced technologies in health services. Mental health remained a consistent theme, with “Depression” and “Mental Health” being prominent. The appearance of “Twitter” as a keyword underscores the specific platforms that researchers are studying within the social media context.

### Dominance of COVID-19 and Advanced Technologies (2020-2023)

In the latest period, the overwhelming influence of the COVID-19 pandemic is evident, with “COVID-19” and “Pandemic” being the most significant keywords. The emergence of “Machine Learning” and “Artificial Intelligence” as top keywords highlights a leap toward more sophisticated technologies in health research. The continuity of “Digital Health” and “Telemedicine” underscores the accelerated adoption of remote and digital health technologies in response to the pandemic. An increasing focus on “Mental Health” and “Anxiety” reflects the impact of challenging times, including the pandemic and social isolation. Another notable development in the period from 2020 to 2023 is the significant increase in publications from China. This rise underscores China’s growing commitment and interest in digital medicine. Even before 2020, there was a steady increase in Chinese research contributions, indicating an increasing prioritization of digital health technologies in Chinese medical research. According to a study by Liang et al [43] published in the *Journal of Healthcare Engineering*, China has a higher publication volume in the medical informatics field’s professional journals compared to Western counterparts, with these publications also demonstrating significant practical application value. This trend is also reflected in the increasing integration of digital technologies into China’s health care system, affecting not only research but also the practical implementation of innovative digital solutions.

### Anticipated AI Focus in Digital Medicine (2024-2026)

The projected keyword trends predicted by Claude 3 Opus signal a stronger emphasis on computational technologies in health care, with “Artificial Intelligence” (17.8%, +10.2%) and “Machine Learning” (12.7%, +5.1%) at the forefront. These trends indicate ongoing innovation in intelligent systems for enhancing health care delivery. “Digital Health” (13.2%, +3.8%) continues to be a mainstay, demonstrating the sector’s growth and the further integration of technology in health care practices. The expected prominence of “ChatGPT” (11.8%, +10.6%) and “Large Language Models” (9.1%, +8.3%) highlights the role of advanced language processing in health communication and data analysis, though the dominance of ChatGPT might get diminished through the raise of proprietary and open-source competitors [16,44,45]. While “COVID-19” (10.2%, –12.7%) remains significant, its reduced trend suggests a shift toward a broader, postpandemic research landscape. Steady interest in “Mental Health” (8.2%, +0.8%) reflects the ongoing commitment to addressing psychological well-being through digital means and maybe in coping with pandemic or postpandemic psychological traumata [46,47].

### Limitations

This study’s findings are subject to several limitations. The keyword generation through the Claude 3 Opus model might not always reflect the full depth of the papers due to the challenges of capturing complex academic content. Consequently, the keywords, though the best according to our benchmark, could include terms with limited relevance. Our analysis is confined to JMIR, which, while a leader in digital health, may have publication and editorial preferences that differ from other journals. As such, the conclusions, particularly regarding trends and foci in digital health research, may not be generalizable across the broader scientific community. Predictive analyses for the period 2024-2026 are inherently speculative and reliant on current trends, which are subject to rapid and unpredictable shifts in the field of digital health technology. These predictions, therefore, should be interpreted with caution. Our focus on JMIR content, predominantly English language and sourced from PubMed, may not represent the global scale of digital health research or capture the full spectrum of contributions from non-English sources and developing regions. This limitation could skew the representation of global research contributions and advancements. Despite these considerations, this study provides a systematic and detailed examination of digital health research evolution over the past 25 years using JMIR as a lens to observe key developments and offering a perspective on the potential future directions of the field.

## Appendix

Multimedia Appendix 1 Top 10 MeSH (Medical Subject Headings) and keywords for given periods.

Multimedia Appendix 2 Claude 3 Opus keyword prediction prompts.

Multimedia Appendix 3 All scripts and data used in this study for reproducibility.

## Abbreviations

AI: artificial intelligence
ARIMA: autoregressive integrated moving average
ES: exponential smoothing
MAE: mean absolute error
MAPE: mean absolute percentage error
MeSH: Medical Subject Headings
MSE: mean squared error
NLP: natural language processing
RMSE: root-mean-squared error
TimesFM: Time Series Foundation Model

## Data Availability

All data generated or analyzed during this study are included in this published paper and in Multimedia Appendices 2 and 3. The raw data used in this study are publicly available through PubMed.
