# Supplementary material for: Decoding the Digital Pulse: Bibliometric Analysis of 25 Years in Digital Health Research Through the Journal of Medical Internet Research
Source: J Med Internet Res. 2024 Nov 15;26:e60057. doi: 10.2196/60057 (PMC11607559; doi:10.2196/60057)
Supplement: Multimedia Appendix 1 [file jmir_v26i1e60057_app1.docx]

| **Period** | **1999 - 2007 (n = 319)** |  |  | **2008 - 2013 (n = 933)** |  |  | **2014 - 2019 (n = 2864)** |  |  | **2020 - 2023 (n = 3952)** |  |  |
| --- | --- | --- | --- | --- | --- | --- | --- | --- | --- | --- | --- | --- |
| **Top** | **KeywordList** | **%** | **Trend** | **KeywordList** | **%** | **Trend** | **KeywordList** | **%** | **Trend** | **KeywordList** | **%** | **Trend** |
| 1 | Health Care And Public Health | 1.3 | New | Internet | 14.9 | New | Internet | 16.4 | 1.5 | Covid-19 | 20.4 | New |
| 2 | Bioethics And Professional Ethics | 0.9 | New | Social Media | 4.5 | New | Ehealth | 11.8 | 7.3 | Mobile Phone | 15.1 | 11.2 |
| 3 | Professional Patient Relationship | 0.6 | New | Ehealth | 4.5 | New | Social Media | 11.8 | 7.3 | Digital Health | 11.1 | 8.4 |
| 4 | Mental Health Therapies | 0.3 | New | Randomized Controlled Trial | 2.4 | New | Telemedicine | 7.5 | 5.4 | Social Media | 11.1 | -0.7 |
| 5 |  | 0 | N/A | Mobile Health | 2.3 | New | Mhealth | 7.2 | 5.4 | Ehealth | 9.9 | -1.9 |
| 6 |  | 0 | N/A | Telemedicine | 2.1 | New | Mental Health | 4.9 | 3.8 | Mhealth | 8.5 | 1.3 |
| 7 |  | 0 | N/A | Depression | 1.9 | New | Depression | 4.8 | 2.9 | Machine Learning | 7.8 | 4.9 |
| 8 |  | 0 | N/A | Mhealth | 1.7 | New | Mobile Phone | 3.9 | 2.5 | Telemedicine | 6.8 | -0.7 |
| 9 |  | 0 | N/A | Systematic Review | 1.6 | New | Randomized Controlled Trial | 3.6 | 1.2 | Artificial Intelligence | 6.4 | 4.8 |
| 10 |  | 0 | N/A | Physical Activity | 1.6 | New | Systematic Review | 3 | 1.4 | Mental Health | 5.9 | 1.1 |
|  |  |  |  |  |  |  |  |  |  |  |  |  |
|  |  |  |  |  |  |  |  |  |  |  |  |  |
| **Period** | **1999 - 2007 (n = 319)** |  |  | **2008 - 2013 (n = 933)** |  |  | **2014 - 2019 (n = 2864)** |  |  | **2020 - 2023 (n = 3952)** |  |  |
| **Top** | **MeSH** | **%** | **Trend** | **MeSH** | **%** | **Trend** | **MeSH** | **%** | **Trend** | **MeSH** | **%** | **Trend** |
| 1 | Humans | 92.2 | New | Humans | 94.5 | 2.4 | Humans | 97 | 2.4 | Humans | 96.3 | -0.7 |
| 2 | Internet | 78.4 | New | Internet | 65.7 | -12.7 | Female | 63.4 | 10.6 | Female | 28.3 | -35 |
| 3 | Female | 36.7 | New | Female | 52.7 | 16.1 | Male | 59.9 | 8.5 | COVID-19 | 24.9 | 24.9 |
| 4 | Male | 33.5 | New | Male | 51.3 | 17.8 | Adult | 39.8 | -4.7 | Male | 22.8 | -37 |
| 5 | Adult | 28.8 | New | Adult | 44.6 | 15.7 | Internet | 38 | -27.7 | Adult | 19.9 | -19.9 |
| 6 | Middle Aged | 23.8 | New | Middle Aged | 36 | 12.2 | Middle Aged | 31.8 | -4.2 | Pandemics | 18.6 | 18.5 |
| 7 | Information Services | 18.8 | New | Young Adult | 20.2 | 18.6 | Telemedicine | 24.8 | 5.4 | Telemedicine | 15.6 | -9.2 |
| 8 | Medical Informatics | 16.9 | New | Telemedicine | 19.4 | 15.6 | Surveys and Questionnaires | 21.6 | 4 | SARS-CoV-2 | 15.4 | New |
| 9 | Patient Education as Topic | 16.3 | New | Aged | 17.9 | 5 | Young Adult | 20.8 | 0.7 | Social Media | 12.9 | -0.6 |
| 10 | Surveys and Questionnaires | 15.4 | New | Surveys and Questionnaires | 17.6 | 2.2 | Adolescent | 17.7 | 1.8 | Surveys and Questionnaires | 11.8 | -9.7 |
