# Supplementary material for: Decoding the Digital Pulse: Bibliometric Analysis of 25 Years in Digital Health Research Through the Journal of Medical Internet Research
Source: J Med Internet Res. 2024 Nov 15;26:e60057. doi: 10.2196/60057 (PMC11607559; doi:10.2196/60057)
Supplement: Multimedia Appendix 2 [file jmir_v26i1e60057_app2.docx]

| **Claude 3 Opus keyword prediction for article titles and abstracts** |
| --- |
| You get a title and an abstract from the Journal of Medical Internet Research article. I want you to determine keywords that describe the article well and return ONLY a JSON list of around 6 to 12 keywords of the most significant keywords for that article (e.g., ["Keyword 1", "Keyword Term 2", "Keyword Term 3", ...]).  Important Note:  - Use concise keywords instead of long terms, e.g., instead of "COVID-19 Pandemic" you have to use the keywords "COVID-19" and "Pandemic" or instead of using "Artificial Intelligence Diagnostic Accuracy" you must use two separate keywords instead: "Artificial Intelligence" and "Diagnostic Accuracy".  - Ensure that each keyword is presented in Title Case, meaning the first letter of each word should be capitalized, except for short prepositions, articles, and conjunctions.  Title: {title} Abstract: {abstract}  List of keywords: |

| **Claude 3 Opus future keyword prediction** |
| --- |
| Given the annual percentage data of the keyword "{keyword}" occurence in the Journal of Medical Internet Research the past years as follows:  {dataframe_with_percentages_of_keyword_occurence_per_year}  Please predict the percentages for the next three years (2024, 2025, and 2026). Do not use any calculation or code, just respond with your general intuition. Return ONLY the predicted values in a JSON dictionary {2024: "Prediction_for_2024", 2025: "Prediction_for_2025", 2026: "Prediction_for_2026"} format without further text, where each key represents a consecutive year starting from 2024, and includes both the year and the predicted proportion percentage. |
